# Comparative overview of emerging RNA viruses: Epidemiology, pathogenesis, diagnosis and current treatment

**DOI:** 10.1016/j.amsu.2022.103985

**Published:** 2022-06-11

**Authors:** Ishani Chakrabartty, Maryam Khan, Saurov Mahanta, Hitesh Chopra, Manish Dhawan, Om Prakash Choudhary, Shabana Bibi, Yugal Kishore Mohanta, Talha Bin Emran

**Affiliations:** a Department of Applied Biology, School of Biological Sciences, University of Science and Technology Meghalaya (USTM), 9th Mile, Techno City, Baridua, Ri-Bhoi 793101, Meghalaya, India; b Department of Biochemistry, Faculty of Life Sciences, Aligarh Muslim University, Aligarh, 202002, U.P, India; c National Institute of Electronics and Information Technology (NIELIT), Guwahati Centre Guwahati, 781008, Assam, India; d Chitkara College of Pharmacy, Chitkara University, Rajpura, Punjab, India; e Department of Microbiology, Punjab Agricultural University, Ludhiana, 141004, Punjab, India; f Trafford College, Altrincham, Manchester, WA14 5PQ, UK; g Department of Veterinary Anatomy and Histology, College of Veterinary Sciences and Animal Husbandry, Central Agricultural University (I), Selesih, Aizawl, India; h Department of Biosciences, Shifa Tameer-e-Millat University, Islamabad, Pakistan; i Yunnan Herbal Laboratory, College of Ecology and Environmental Sciences, Yunnan University, Kunming, 650091, China; j Department of Pharmacy, BGC Trust University Bangladesh, Chittagong, 4381, Bangladesh; k Department of Pharmacy, Faculty of Allied Health Sciences, Daffodil International University, Dhaka, 1207, Bangladesh

**Keywords:** RNA virus, COVID-19, SARS, MERS, Emerging diseases

## Abstract

From many decades, emerging infections have threatened humanity. The pandemics caused by different CoVs have already claimed and will continue to claim millions of lives. The SARS, Ebola, MERS epidemics and the most recent emergence of COVID-19 pandemic have threatened populations across borders. Since a highly pathogenic CoV has been evolved into the human population in the twenty-first century known as SARS, scientific advancements and innovative methods to tackle these viruses have increased in order to improve response preparedness towards the unpredictable threat posed by these rapidly emerging pathogens. Recently published review articles on SARS-CoV-2 have mainly focused on its pathogenesis, epidemiology and available treatments. However, in this review, we have done a systematic comparison of all three CoVs i.e., SARS, MERS and SARS-CoV-2 along with Ebola and Zika in terms of their epidemiology, virology, clinical features and current treatment strategies. This review focuses on important emerging RNA viruses starting from Zika, Ebola and the CoVs which include SARS, MERS and SARS-CoV-2. Each of these viruses has been elaborated on the basis of their epidemiology, virulence, transmission and treatment. However, special attention has been given to SARS-CoV-2 and the disease caused by it i.e., COVID-19 due to current havoc caused worldwide. At the end, insights into the current understanding of the lessons learned from previous epidemics to combat emerging CoVs have been described. The travel-related viral spread, the unprecedented nosocomial outbreaks and the high case-fatality rates associated with these highly transmissible and pathogenic viruses highlight the need for new prophylactic and therapeutic actions which include but are not limited to clinical indicators, contact tracing, and laboratory investigations as important factors that need to be taken into account in order to arrive at the final conclusion.

## Highlights

• Recently published review articles on SARS-CoV-2 have mainly focused on its pathogenesis, epidemiology and available treatments.
• The pandemics caused by different CoVs have already claimed and will continue to claim millions of lives.
• This review focuses on important emerging RNA viruses starting from Zika, Ebola and the CoVs which include SARS, MERS and SARS-CoV-2.
• Globally, numerous studies and researchers have recently started fighting this virus.

## 1 Background

The endless expansion of the human population is meticulously linked to globalization, trade, and habitat fragmentation which thereby promote interaction between people, domestic animals, and wildlife populations. This poses a risk of contracting various microbes to which they had not been previously exposed. In the recent past, the increasing human contact with wildlife has resulted into a number of pandemics that originate from wildlife reservoirs [1]. In particular, RNA viruses are the key zoonotic agents, originating from wildlife, and causing deadly pandemics around the world among all potential pathogens that are involved in inter-species transmissions. According to the studies conducted in the last decade, RNA viruses are considered as main etiological agents of human pathogens, inhabiting 44% of all emerging contagious diseases [2]. RNA viruses have greater ability to infect new host species because of their extremely shorter generation time and rapid evolutionary rate. Mutation rate of RNA viruses is known to occur at around six times higher magnitude than those of their cellular hosts and their mutability can exceed up to five orders of magnitude than that of some DNA viruses [3,4]. The summarized information which discussed extensively in this review is shown in Table 1. A comparison between the mutation capabilities of different microbes has been illustrated in Table 2.

**Table 1:** Summarized information discussed extensively in this review.

Organism	Group and Virology		Epidemiology		Pathogenesis		Management and Treatment
RNA Viruses	Zika; ss, + strand RNA genome encoding 7 non-structural proteins and 3 structural proteins		Started in Brazil in 2007; on decline since 2019. More than 200,000 cases		Transmitted by mosquito bites or sexual intercourse; humans – incidental hosts. Shed in the urine during illness		No proper antiviral or vaccine available; prevention of mosquito bites, awareness and knowledge of the disease practiced
	Ebola; ss, - strand RNA genome encoding structural and non-structural proteins		1st outbreak in Zaire in 1976; 2nd outbreak in Africa in 2014. On decline since 2019 but cases are still reported		Transmission by formite, aerosols or direct contact; no report through sexual intercourse. High rate of mortality		No antiviral or vaccine available; ringworm vaccine found to be effective. Preventive measures and awareness programmes undertaken
	SARS		1st reported in China in 2002		Mostly like respiratory illness; inter (between man and camel) and intra-human transmission reported		Sanitization, masking, social distancing
	MERS		1st reported in Oman in 2012; 27 countries affected				
	COVID-19		1st reported in China in 2003 during Spring festival (not severe). Next outbreak at same time in 2020 in China; major countries affected – cases still being reported		Mostly like respiratory illness; inter (between bat and man) and intra-human transmission reported through direct contact		Diagnosis through RT-PCR, CRISPR-based assays. Vaccines from Pfizer, Serum Institute of India, Bharat Biotech available – some more vaccines are underway
**COVID Mutations**							
αβ variant in 2019–2020; origin China		Δ variant in 2020–2021; origin United Kingdom		Omicron variant in 2021–2022; origin South Africa		New variant (deltacron???) in 2022–2023??; origin?

**Table 2:** Comparison of mutation rates of different microorganisms.

Organism	Group	Evolutionary mutation rate (mutations/site/year)	Reference
RNA Viruses	Zika	4 × 10^−3^	[5]
	Ebola	Unknown	
	SARS	0.80-2.38 × 10^−3^	[6]
	MERS	1.12 × 10^3^	[7]
	COVID-19	Unknown	
Bacteria	*Escherichia coli*	2 × 10^−10^	[8]
Fungi	*Saccharomyces cerevisiae*	3.3 × 10^−10^	[9]
Protozoa	*Plasmodium falciparum*	1 × 10^−9^	[10]

**Table 3:** Total number of confirmed, recovered and death cases worldwide due to COVID-19 pandemic according to WHO report as on November 08, 2021.

Country	Total cases		Total deaths	Active cases	Critical/serious cases	Continent
	Confirmed	Recovered				
World	250,892,010	227,118,890	5,068,937	18,704,183	76,410	–
United States	47,345,192	37,351,695	775,346	9,218,151	11,206	North America
India	34,374,455	33,763,237	461,347	149,871	8,944	Asia
Brazil	21,880,439	21,069,794	609,484	201,161	8,318	South America
Russia	8,834,495	7,587,560	248,004	998,931	2,300	Asia
France	7,219,681	6,978,133	117,965	123,583	1,049	Europe
Turkey	8,259,503	7,737,259	72,314	449.340	1,440	Europe
UK	9,333, 891	7629,990	141,862	1,562,039	1,026	Europe
Spain	1,715,700	N/A	46,646	N/A	2,371	Europe

**Fig. 1:**
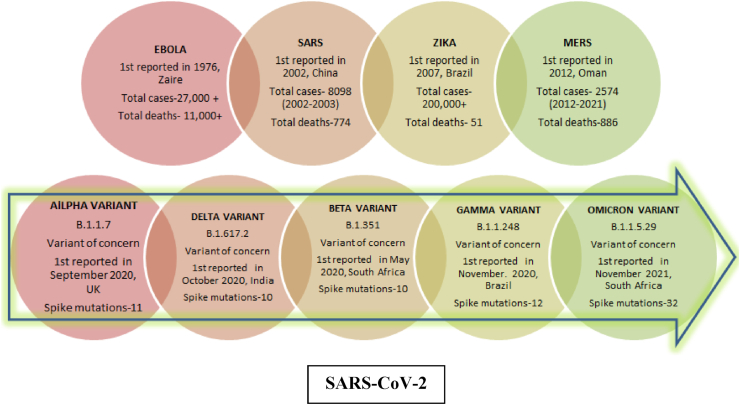
Timeline of emerging RNA viruses.

With 2-3 novel viruses being discovered every year, RNA viruses are considered as the most common class of pathogens and causative agents behind the currently emerging human outbreaks [11]. Vast amount of literature is available on a number of emerging interspecies-transmitted RNA viruses, such as H1N1 influenza, the highly pathogenic H5N1 avian influenza *Ebola* viruses, *Zika* (ZIKV) viruses, the Severe Acute Respiratory Syndrome Coronavirus (SARS-CoV), Middle East Respiratory Syndrome Coronavirus (MERS-CoV) and the most recent one i.e. Severe Acute Respiratory Syndrome Coronavirus (SARS-CoV-2) [12,13]. A timeline of when these viruses emerged and some important information related to it has been summarized in Fig. 1.

In this review, we summarize the epidemiology, virology, clinical features, transmission and current treatment strategies of some of the highly pathogenic RNA viruses’ viz. Ebola, Zika, SARS, MERS and SARS-CoV-2. Herein, special emphasis has been given to SARS-CoV-2 due to prevailing threatening conditions worldwide.

## 2 Viruses

### 2.1 Zika virus

*Zika* virus or ZIKV is a positive-strand (+) RNA virus, belonging to the virus family flaviviridae. This virus is primarily transmitted by the bite of *Aedes* mosquito (*A. aegypti* and *A. albopictus*), which are known to bite mostly during the day and sometimes, during night. First identified in the year 1947 in Uganda in monkeys, the virus was later found in humans in Uganda and United Republic of Tanzania in the year 1952 [14,15]. *Zika* virus is believed to have 2 lineages: Asian and African; Asian strain is believed to have evolved around 1928. Phylogenetically, *Zika* virus infection occurring in Americans is ∼89% similar to the African genotype; however, the virus causing the outbreak in 2013–2014 in French Polynesia is more similar and related to the Asian lineage [[16], [17], [18], [19]].

#### 2.1.1 Epidemiology

Initially, outbreak of *Zika* virus disease was recorded from the Island of Yap (Federated States of Micronesia) in 2007. This was trailed by a large outbreak of *Zika* virus infection in French Polynesia in 2013 and other territories in the Pacific. Though initial ZIKV infections are asymptomatic, there are strong evidences of these infections to be related to microcephaly and other genetic defects like limb deformities, retinopathy etc. [[20], [21], [22]]. A large outburst of rash illness was reported in Brazil in March 2015 that was rapidly identified as *Zika* virus infection, and in July 2015, it was found to be linked with Guillain-Barré syndrome [14,[23], [24], [25]]. In February 2016, WHO declared ZIKV pandemic as a public health emergency of international concern (PHEIC); the enormity of the pandemic was accelerated several fold due to the Olympic games being scheduled in Rio de Janerio, Brazil in November 2016 [[25], [26], [27]]. In all, more than 85 countries and territories had reported evidence of mosquito-acquired *Zika* infection; the greatest impact of the virus was found in Brazil. More than 200,000 cases of *Zika* virus disease have been estimated to have occurred and approximately 8600 babies have been born with malformations in that country.

The epidemic has since declined, and till May 2019 no countries had reported active outbreaks of *Zika* virus. According to Centers of Disease Control and Prevention (CDC), around 25 locally acquired cases were reported in the US in 2021 by serological testing that detected antibodies against ZIKV – since antibodies can persist in the bloodstream for years, serological testing does not confirm a recent infection (https://www.cdc.gov/zika/reporting/2021-case-counts.html). In 2021, cases of *Zika* virus have begun surfacing in India in states like Kerala, Maharashtra, and Uttar Pradesh. Though it has not reached a severe condition, red alerts have been issued by the respective state governments across the country (https://www.who.int/emergencies/disease-outbreak-news/item/zika-virus-disease-india) [28]. *Zika* virus and the mosquitoes that transmit it have still not been vanished and a low risk of currently undetected transmission still prevails in our body. Therefore, people should follow strict vigilance and caution, especially women who are pregnant or considering pregnancy (https://www.bcm.edu/departments/molecular-virology-and-microbiology/emerging-infections-and-biodefense/zika) [29].

#### 2.1.2 Virology

*Zika* virus is an encapsulated, icosahedral virus having a non-segmented single-stranded (ss), positive RNA (+) genome; hence, it can be directly transcribed and translated into viral proteins – this ss RNA genome of *Zika* virus encodes for 7 non-structural proteins and 3 structural proteins – one of which encapsulates the virus [30]. This encapsulation protein or glycoprotein binds to the host cell to facilitate the host-pathogen interaction and initiate endocytosis. The RNA genome forms a nucleocapsid along with numerous copies of the 12 kDa capsid protein which in turn, is enclosed within a host-derived membrane that is modified using 2 viral glycoproteins. Viral genome replication consists of making a double-stranded (ds) RNA from the ss (+) RNA genome, and further produce viral mRNAs and new ss RNA (+) genomes [[30], [31], [32]]. After around 6 h of *Zika* virus infection, the mitochondria and vacuoles of the infected cells starts swelling up rapidly; even the endoplasmic reticulum starts forming vacuoles. Eventually, this profuse swelling leads to paraptosis – a kind of programmed cell death [29,32,33].

#### 2.1.3 Transmission and pathogenesis

*Zika* virus infection is most commonly transmitted through mosquito bite; initial infections tend to occur on human skin affecting dermal fibroblasts, keratinocytes, etc. The pathogenesis of the virus is hypothesized to continue till the virus spreads to lymph nodes and the bloodstream. Ideally, flaviviruses replicate in the cytoplasm but the prevalence of *Zika* antigens in infected cell nuclei suggests that the process occurs in the nuclei of the host cell [34,35]. *Zika* is also known to be transmitted by sexual intercourse between asymptotic males and uninfected females, and vice versa. There are experimental evidences in mice that suggest that ZIKV infects placental cells via *trans*-placental route that hinders the intrauterine growth. In 2016, it was reported that 29% of babies born to ZIKV infected mothers had developmental abnormalities that confirmed this route of transmission known as vertical transmission [36,37]. An important thing worth noting is that people having history of other flavivirus infections are at a higher risk of acquiring ZIKV infections [38]. In addition, *Zika* virus can be transmitted through blood transfusions and sexual intercourse (oral, vaginal, or anal) with an infected person [39].

*Zika* virus generally follows the salivatic transmission cycle. It is as an arbovirus, transmitted from one vertebrate to another by a mosquito bite. Like most viruses of this family, it exists as immature (non-infectious), mature (infectious), and fusogenic (host membrane binding) states. Humans are only the incidental hosts in the lifecycle of the virions; however, monkeys and apes can also serve as host. Some studies have revealed that antibodies against *Zika* were found in the bloodstream of sheep, elephants, and goats, suggesting these animals serve as possible host as well. When an *Aedes* mosquito ingests blood containing *Zika* virus after stinging an infected person, the virus starts replicating in the epithelial cells of midgut of the mosquito and travels to its salivary glands. After approximately 10 days (incubation period), the saliva becomes infected; thus, the mosquito becomes a vector, armed with the ability of infecting another human. Moreover, *Zika* virus can also lead to the autophagy of host cells; once replication is complete, the virus spreads homogenously and leads to fatal condition by spreading to distant organs like the nervous system, through the bloodstream [35,40].

It is interesting to note that the RNA of the virus is generally shed in around 60% of infected men during first 30 days of illness. However, subsequent decrease is observed in the amount of viral RNA being shed after 3–9 months of illness; a similar pattern of viral RNA shedding was observed in the urine of infected males [41,42]. The virus is known to have a long latent period in the semen and hence, is one of the major factors responsible for sexual transmission [43].

#### 2.1.4 Management and treatment

ZIKV has a deleterious effect on pregnancy as it can only be first identified during the first stage of infancy. However, only around 7% of the fetuses and infants were infected from ZIKV infected mothers – major defects in infants were observed when the pregnant women were infected during early pregnancy [44]. A major limitation in the management of this virus is that the viral infection is mostly asymptomatic or resembles that of a mild dengue fever; diagnostic measures and therapies are also not ideal, making diagnosis and ascertaining the timing of infection problematic [43,45]. It presents both clinical as well as diagnostic challenge since the symptoms are quite similar to other arboviral diseases, with no specific and commercial serological tests available to date. A cross-reactive DENV serology (IgG or IgM) during ZIKV infection has also been reported in some cases, which may lead to improper diagnosis and give false positive reports [46].

Though no proper treatment of ZIKV is available, a DNA vaccine against the virus is in its initial phase of testing; the initial safety and immunogenicity of a DNA vaccine, encoding consensus ZIKV pre-membrane and envelope antigens is being delivered by means of electroporation [47]. Most importantly, prevention is better than cure. As there are no antivirals or vaccines available, hence prevention messages, including recommendations on avoiding mosquito bites and education on sexual ZIKV transmission, is currently the best strategy to combat this virus globally [43,48].

### 2.2 Ebolavirus

*Ebolavirus* or *Zaire ebolavirus* (EBOV) belongs to a family of viruses known as *Filoviridae*, which are ideally long, filamentous, branched or unbranched virus particles, containing single-stranded (ss), negative (−) sense RNA genome, enveloped within a lipid membrane (https://www.bcm.edu/departments/molecular-virology-and-microbiology/emerging-infections-and-biodefense/ebola-virus) [49]. The virus family *Filoviridae* comprises of three genera: *Cuevavirus*, *Marburgvirus*, and *Ebolavirus*; around 6 species have been recognized from the genus *Ebolavirus*: Zaire, Bundibugyo, Sudan, Taï Forest, Reston and Bombali. It is one of the known species of the genus *Ebolavirus* that is the causative agent of the fatal hemorrhagic fever in humans and other mammals - this disease is known as Ebola virus disease (EVD). The virus for the first time showed fatality in 1976 in 2 parallel outbreaks - one in now Nzara, South Sudan, and the other in Yambuku, the Democratic Republic of the Congo (DRC); the latter occurred in a village near the Ebola River, from which the disease gets its name. *Zaire ebolavirus* was originally named for Zaire (now DRC), the country where it was initiated. It was at first suspected to be a new “strain” of the closely related *Marburgvirus*; however, the virus was renamed “*Ebolavirus*” in 2010 to avoid confusion [50,51].

The natural reservoir of EBOV is found to be bats (belonging to order Chiroptera), particularly fruit bats, that serve as the intermediary hosts for this virus; hence it is called a zoonotic pathogen [52]. End hosts are generally humans, apes and other primates and the virus is believed to be transmitted from animals to humans by the exchange of body fluids; however, it is surprising to note that the virus has never been isolated from bats [52,53]. *Ebolavirus* outbreaks tend to occur in specific climatic conditions - when temperatures are lower and humidity is higher than usual, as observed during the outbreaks in Africa. An important point to be kept in mind is that even after a person is cured from the acute phase of the disease, *Ebolavirus* tends to survive and reside for months in certain specific organs such as the eyes and testes [[54], [55], [56]].

#### 2.2.1 Epidemiology

EVD is one of the deadliest viral diseases that first broke out 1976 – in this very first outbreak, around 300 people were infected; mortality rate was 88% in Zaire and 53% in Sudan. In 1989, Reston *Ebolavirus* was discovered in monkeys that were transported from Philippines to USA for research purpose; at least 4 humans were infected, though it killed none [49]. Later on, scientists confirmed that the virus had spread in the entire monkey population through aerosols i.e., droplets in the air (aerosolized transmission) in the facility. However, such airborne transmission is not considered as a significant contributor to the human outbreaks of *Ebola.* The discovery of the Reston virus in the monkeys from the Philippines revealed that *Ebola* was no longer confined to African settings, but had spread to the Asian subcontinent as well (https://www.cdc.gov/vhf/ebola/history/summaries.html) [57,58].

In 2014, another *Ebola* outbreak occurred in Africa, for the first time in West Africa. Between 2013 and 2016, this virus was found to have the substantial epidemic potential and forced WHO to declare it as PHEIC – the outbreak was at an unprecedented scale with over 28,000 confirmed cases and more than 11,000 deaths – something that totally crippled the economy of Africa [59,60]. Research has it that EVD had mostly affected the economically deprived countries that lack proper infrastructure, administration and medical facilities [57,61,62]. Ebola virus disease in 2014 was recorded as the biggest pandemic of all times and in 2018, another *Ebolavirus* outbreak occurred in DRC again, which ranked as the 2nd pandemic; though the latest COVID-19 pandemic is has surpassed those numbers. However, by June 2019, the pandemic was controlled immensely though it claimed as many as 1400 human lives (the original number is believed to be higher) – a wide partnership of government and local agencies worked tirelessly to curtail the virus enormously [49]; however, as of the present date, new cases of EVD continue to be reported from African subcontinent (https://www.afro.who.int/health-topics/disease-outbreaks/outbreaks-and-other-emergencies-updates) [63]. Despite this, on May 3, 2021, the DRC Ministry of Health and WHO declared the end of Ebola outbreak in the North Kivu province, DRC (https://www.cdc.gov/vhf/ebola/outbreaks/drc/2021-february.html) [64].

#### 2.2.2 Virology

Like all viruses belonging to the *Filovirus* family, *Ebola* too is a pleomorphic, ss, (−) RNA virus; the genome is 19 kb long and encodes both structural and nonstructural proteins – the structural proteins include nucleoprotein, GP and viral matrix proteins VP24 and VP40, whereas the non-structural proteins are viral polymerase and other matrix protein viz. VP35 and VP30. Though, *Ebolavirus* is considered to be very similar to *Marburgvirus*, there are subtle differences between them - the GP open reading frame of *Ebolavirus* gets translated into 2 gene products - a soluble 60- to 70-kDa protein (sGP) and a full-length 150- to 170-kDa protein (GP); the latter gets inserted into the viral membrane through transcriptional editing, unlike *Marburgvirus* [[65], [66], [67]]. It is reported that a very low infectious dose (1–10 virions) is sufficient to cause infection in non-human primates, from which transmission can occur by aerosols. The incubation period of the virus lasts from 2 to 21 days – during this period, the EBOV replicates rapidly – the primary and secondary replication occurs in the inclusion bodies. New viral genomes are organized into nucleocapsids and packaged into progeny viral particles – a process mediated by VP40 [58,65,68,69]. Once the viral particles are formed, the host immune system is down regulated to a large extent - VP35 and VP24 are strong inhibitors of interferon production and interferon signaling, respectively. In addition, VP35 has numerous functions that hinder with the early innate host response [58,70].

Initial EVD infection presents itself with non-flu-like symptoms including fever, myalgia and malaise – these symptoms surface within 8–21 days of infection. As the infection advances, the patient exhibits severe bleeding and coagulation abnormalities, including gastrointestinal bleeding, rash, and a series of hematological irregularities, such as lymphopenia and neutrophilia, followed by damage to the liver, pooled with massive viremia, leading to disseminated intravascular coagulopathy. Eventually, the EBOV infects micro-vascular endothelial cells and compromises vascular integrity. The last stage of *Ebolavirus* infection mostly include diffuse bleeding, and hypo/hypertensive shock that account for majority *Ebolavirus* fatalities [65,71,72].

#### 2.2.3 Transmission and pathogenesis

In order to control the outbreak and determine appropriate health-care practices, it is very important to determine the possible modes of transmission of *Ebolavirus* between people. The virus is found in a variety of body fluids including saliva, blood, breast milk, faeces, and even semen of infected males; hence there are numerous routes of viral transmission like formite, aerosols and most importantly, direct contact [72]. However, the CDC had communicated on a global platform that EBOV is transmitted only through direct contact of mucus membrane or infected blood and body fluids through broken skin and contaminated needles, surgical instruments or from infected animals/vectors or carriers [73]. Some intricate investigations have also revealed that infections also occur through intimate exposure of care-giving and burial preparation [58,74].

High level of EBOV viral particle formation, coupled with the systemic dissemination of vital cell types, along with the suppression and over-activation of the immune system, form a complex pathogenesis of the EVD. The virus, upon reaching the regional lymph nodes, causes lymphadenopathy and hematogenous spread to the liver and spleen that stimulate an active inflammatory response. Discharge of certain chemical mediators of inflammation viz. cytokines and chemokines cause a downregulation of the immune response. This occurs by disrupting the vasculature system coordination, and eventually causing disseminated intravascular coagulation along with multiple organ dysfunction [71,75]. In the absence of extensive care and proper treatment, these processes lead to overall thrombocytosis and multiple organ failure – this can turn fatal and cause death within 10 days of onset of infection [76].

Till now, no clear information is available on the transmission of EBOV by sexual intercourse – whether oral, anal or others. An infected male's semen showed the presence of viral RNA after 3 months of onset of infection [71]; however, it is to be kept in mind that the detection of viral RNA in any body fluid like sweat, blood or even semen does not necessarily indicate that the body/discharged fluid is infectious in nature [77]. Like ZIKV, the shedding of viral RNA in the semen of infected male declined within the increase in duration of infection. Despite the fact, very rare cases of EVD have been related to sexual transmission, intense research is essential to investigate whether infectious virus are found in vaginal fluid or other body fluids even after recovery. Furthermore, additional testing of body fluids, both in male and female survivors, is a requisite to reach a final conclusion [77,78].

#### 2.2.4 Management and treatment

*Ebolavirus* is known to have the potential of causing 90% mortality. Hence, it also possesses the risk of causing further outbreaks in the near future. The latest outbreak of EBOV in October 2021 in Beni Health Zone, North Kivu Province, DRC highlights the complex challenges that are present to curtail the spread of the virus in a geographically remote and isolated region that is already lacking huge amount of facilities, coupled with mistrust and misinformation from the government as well as non-government authorities [49,64]. It is a lesser-known fact that Ebola was considered a neglected disease until the 2014 outbreak; hence, research on the anti – viral therapy against this virus is seriously lacking. There is no cure or established drug, approved vaccine or therapy available till date to treat this viral disease; though an unlicensed vaccine was found to be effective during the 2014 pandemic – as many as 120,000 people were vaccinated using the ringworm vaccination during the outbreak in 2018.

Research carried out by some groups revealed that passaging the *Zaire ebolavirus* in guinea pigs may lead to changing the infectivity of the virus - it is even possible that with time and evolution of EBOV in individuals of the same species, new variants may arise which are more or less virulent to humans. Overall, the ecology of the individual animal itself determines the risk of infection in humans that reflect the heritage of the virus and may even contribute to the sporadic nature of EBOV outbreaks [79]. The strategic line of treatment offered to infected patients include symptomatic and supportive care, such as hydration, replacement of electrolytes, nutritional support, maintaining oxygen status and blood pressure and treating other infections. However, not everything is as bleak as it seems - there are two notable Ebola vaccine efforts viz. cAd3-ZEBOV and rVSV; both vaccines have shown positive results in non-human primates, but the effectiveness in humans requires further studies [60,79]. The old proverb “Prevention is better than cure” stands very true in case of such viral diseases – the suspected and confirmed EVD patients are required to be isolated immediately and quarantined. According to WHO and CDC recommendations, infected women – even lactating mothers are required to avoid any close contact with their infants and nutrition should be provided in other ways. Sexual intercourse should also be avoided till the semen or vaginal discharge tests negative for viral RNA [76].

An in-depth knowledge of the mechanisms underlying *Ebolavirus*-induced cytopathic effects has enabled the smooth process of vaccine and antiviral therapy development, which in turn, has provided important information about pathogenesis and the immune response against the virus [59,65]. Since 2014, clinical care for EVD has progressed with the advent and development of new therapeutic agents, patient cubicles, and aggressive supportive care. However, due importance should be given to the implementation of basic pillars of outbreak response which include effective contact tracing and prevention of community engagement. This in turn can lead to rapid identification of people who are ill and facilitate the provision of early clinical care subsequently improving and increasing the likelihood of survival [80]. It has been more than 2 years since the last Ebola outbreak in the African continent but the region has not ceased to report new cases and hence, is still a boiling frog! A deliberate as well as selfless scale-up of involvement in support of and in collaboration with the on-ground partners, including the WHO and DRC government would ensure a seamless effort that is in accordance to the communities' and partners’ needs and thus, and thus can reduce the number of new cases drastically. Such a set-up would go a long way in winning the hearts, minds and cooperation of the local population who are actually the true victims or sufferers in the pandemic [81].

### 2.3 Coronaviruses

Coronaviruses (CoVs; subfamily *Coronavirinae*, family *Coronaviridae*, order *Nidovirales*) comprises a group of highly diverse, enveloped, positive-sense (+), single-stranded (ss) RNA viruses that lead to respiratory, enteric, hepatic and neurological implications of different intensity in a broad range of animal species, including humans [82]. In 1962, CoVs have been defined as a respiratory tract virus as the samples were collected from the individuals who demonstrated symptoms of respiratory tract infection [83]. These viruses comprise the largest genome (30,000 bases of positive-sense RNA) set up found in any RNA virus. However, CoVs, like other RNA viruses, mutate less rapidly as a consequence of the proof-reading nature of RNA polymerases. Thus, minor mutations can develop abruptly and persist as a founder effect [1]. CoVs are a vast family of viruses found in different animal species, including camels, cattle, cats, and bats. CoVs are subdivided into four genera: alpha-CoV, beta-CoV, gamma- CoV and delta-CoV [84]. Gamma and delta CoVs largely infect birds, although some cases of infection are also reported in mammals. While alpha and beta CoVs are known to infect humans and animals only. Among all CoVs, SARS-CoV (beta coronavirus), 229E (alpha coronavirus), HKU1 (beta coronavirus), NL63 (alpha coronavirus), OC43 (beta coronavirus) and MERS-CoV (beta coronavirus) can cause severe infections in humans. However, beta-CoVs are the most lethal ones as this group contains some of the highly pathogenic viruses found in humans [[84], [85], [86]]. It is a rare phenomenon when animal CoVs can infect humans and human chain transmission occurs such as witnessed during epidemics like MERS, SARS, and COVID-19 (*Coronavirinae in ViralZone*, availaible online: https://viralzone. expasy.org/785) [[87], [88]]. Though CoVs infecting humans have been identified from decades, their clinical significance and epidemics were not recognized until the recent outbreaks of SARS and MERS [90].

#### 2.3.1 Severe acute respiratory syndrome (SARS)

SARS is a respiratory illness caused by the zoonotic RNA human coronavirus (CoV) group 2b, SARS-CoV [91]. In February 2003, a novel CoV was confirmed to be the causative agent for SARS, thus referred to as SARS-CoV [91]. This outbreak was originated in Guangdong Province in the People's Republic of China in 2002, which rapidly spread to Hong Kong and subsequently to 33 other countries over 5 continents. While its intermediary animal host is still unclear, animal to human transmission is believed to have originally occurred through the masked palm civet, that has substantial human interaction in outdoor Chinese markets [92]. During the SARS outbreaks in late 2003 and early 2004, three of the four patients were directly or indirectly in contact with palm civets [93]. However, sequence analysis revealed that the SARS-CoV-like virus had not been circulating amongst masked civets in markets for long. CoVs that are highly similar to SARS-CoV were isolated in horseshoe bats in 2005 [93]. The SARS-like CoVs in bats pooled around 88–92% sequence homology with humans or civet isolates and the figures suggest that bats could act as a natural reservoir of a close predecessor of SARS-CoV [93].

##### 2.3.1.1 Epidemiology

The index case causing subsequent outbreaks of SARS in Hong Kong and many other countries was found to be a 64-year-old nephrologist, who came from southern China to Hong Kong on February 21, 2003 [91]. Subsequently, 16 hotel lodgers and their visitors were infected by the physician while staying or visiting friends on the same floor of the hotel M, where the nephrologist had stayed. These visitors spread the infection to 29 countries and regions through international flight travel. A total of 8098 cases and 774 deaths, with mortality rate of 9.6% by the end of the epidemic in July 2003, were reported [94]. SARS appears to have spread by close human contact via droplet transmission or fomite [95]. The highly contagious nature of SARS was revealed by the cluster event at the Prince of Wales Hospital (PWH) in Hong Kong, where 138 individuals (mostly health care workers; HCWs and previously healthy) were found positive within 2 weeks of exposure to one single SARS positive person (a visitor of hotel M). This patient was diagnosed with community-spread pneumonia and hospitalized in a general medical ward [96]. The main cause of this super-spreading event includes the use of a common jet nebulizer for delivering bronchodilator to the index case, congestion and poor ventilation in the hospital ward [97]. SARS-CoV was also found in the respiratory secretions, faeces, urine and tears of infected individuals [98]. Disease was more fatal in the elderly, with a mortality rate of greater than 40% in patients over 60 years of age. Since 2004, no more infections have been detected, and the SARS pandemic was declared to be ended [99].

#### 2.3.2 Middle East respiratory syndrome (MERS)

After ten years of the emergence of SARS-CoV, another highly infectious CoV, Middle East respiratory syndrome Coronavirus (MERS-CoV) had emerged in the Middle East countries. The causative virus that was originally called human CoV-Erasmus Medical Centre/2012 (HCoV-EMC/2012) was later renamed as Middle East Respiratory Syndrome Coronavirus, MERS-CoV [100]. At the outset, it was isolated from a patient who died because of severe respiratory illness in Saudi Arabia in 2012 [101,102]. Many researchers have reviewed the history of MERS in the literature [103,104]. MERS-CoV is a zoonotic disease that is transmitted from animals-to-humans. Furthermore, MERS-CoV can be transmitted to humans *via* both animals and humans and bats are likely to be the main mammalian reservoir [104]. Literature suggests that Dromedary camels, the intermediate hosts for MERS-CoV, have been involved in direct and indirect transmission to humans, although the exact mode of transmission is still not known [103].

##### 2.3.2.1 Epidemiology

The first study showing camels as a potential source and reservoir of MERS-CoV was from Oman and based on positive serological results obtained from 50 serum samples of dromedary camels (*Camelus dromedaries*) that showed a high titer of neutralizing antibodies against MERS-CoV. Furthermore, in a study of two human cases infected by MERS-CoV in Qatar; out of the 14 confirmed dromedary camels, three tested positive for MERS-CoV [105]. Interestingly, an investigation showed that camels may have acquired MERS-CoV from bats in sub-Saharan Africa, with successive import of camels to the Arabian Peninsula [106]. For clear understanding of epidemiology and evolution of MERS-CoV in different hosts, identification of the major route of animal-to-human transmission is important [107]. However, the consecutive epidemics of MERS indicate that this CoV has spread to different parts of the world predominantly *via* inter-human transmission. Human-to-human transmission was confirmed by the fact that secondarily infected individuals had come in close proximity of a primarily infected individual, which mostly comprised family members, HCWs and people who shared the hospital room or visited the patients [108]. The global report by WHO has shown that between 2012 and March 2021, there were 2,574 laboratory-confirmed cases of MERS, including 886 associated deaths (case-fatality rate = 37.2%) (https://applications.emro.who.int/docs/WHOEMCSR415E-eng.pdf?ua=1). Due to frequent air travel, the virus has spread from the Middle East to 27 countries in total and at some place's chains of human-to-human infections have also been found, e.g., in the Republic of Korea. The first Korean patient infected by MERS-CoV was diagnosed on 20 May 2015, after he returned from Qatar [109]. Owing to the secondary mode of transmission, 186 people of Korean population were found to be infected with MERS-CoV in a short period of time. Phylogenetic analysis confirmed that the MERS-CoV isolate found in the Korean patient is closely associated to the Qatar strain. Of the total cases which have been confirmed from 27 countries, majority of them (i.e, 1,901) were reported from Saudi Arabia [110].

#### 2.3.3 SARS-CoV-2

The new decade of the 21st century began with the emergence of a new coronavirus, SARS-CoV-2, which is currently a serious community health problem and is the focus of global attention [111]. The site of origin of SARS-CoV-2 was Huanan Seafood Market, in Wuhan State of Hubei Province in China (Seven days in medicine: 8–14 Jan 2020. *BMJ*. 2020;368:m132. Published 2020 Jan 16, https://doi.org/10.1136/bmj.m132), where livestock animals are traded. The index case of the COVID-19 epidemic was discovered with unusual pneumonia on December 12, 2019, and subsequently 27 viral pneumonia cases, with 7 being serious, were formally announced on December 31, 2019. In addition, possible influenza and other CoVs were ruled out by laboratory testing in the reported cases. Chinese government declared on January 7, 2020 that a new type of Coronavirus (novel Coronavirus, nCoV) was isolated which was further named as SARS-CoV-2 by WHO on January 12 (Imperial College London. Report 2: estimating the potential total number of novel CoV cases in Wuhan City, China. Jan 2020. https://www.imperial.ac.uk/mrc-globalinfectiousdisease- analysis/news--Wuhan-coronavirus). The gain of infectious cases in Wuhan city and internationally after even complete lockdown and evacuation of the cases in China are indicative of secondary transmission chain from human-to-human. Fresh cases are still being identified, predominantly in Asian countries and in other countries such as the *trans*-oceanic USA and France. The median estimated incubation period of the virus is approximately of 5.5 days, ranging from 0 to 14 days [112]. Reports from China and Italy confirm that about 80% of patients are asymptomatic and the median patient age is less than 60 years. The most common symptoms of COVID-19, a disease caused by SARS-CoV-2 include fever, weakness, sore throat and cough [113]. More than 50% of the patients report shortness of breath, with few developing acute respiratory distress syndromes. After septic shock, refractory metabolic acidosis and coagulation dysfunction can occur that can lead to sudden death. Similar to SARS and MERS, human-to-human transmission in COVID-19 occurs primarily through direct contact or air droplets [114]. The high risk of transmission is when a person comes in contact with an infected person standing within approximately 1 m distance (about 3 feet); however, the maximum distance is still undetermined [112].

##### 2.3.3.1 Epidemiology

**Fig. 2:**
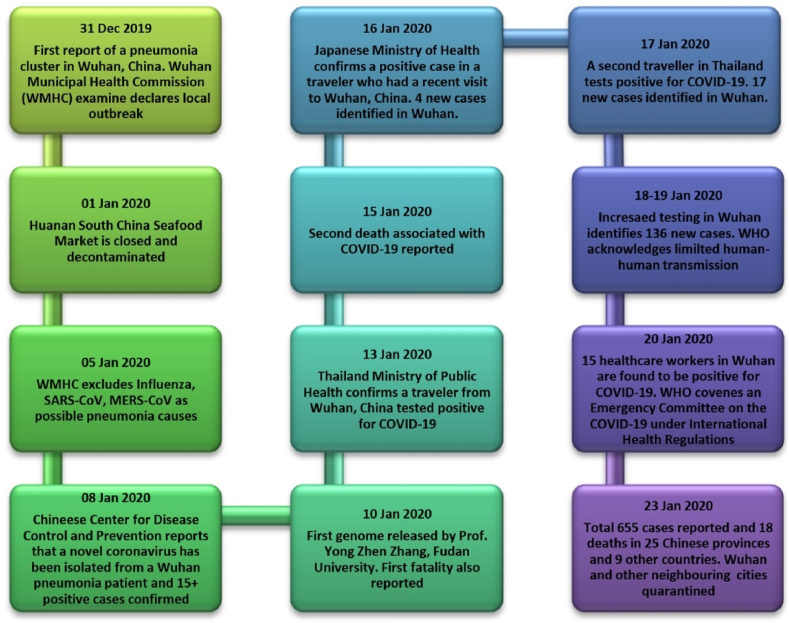
Chronology of events reported for COVID-19 outbreak (Source: WHO).

Similar to the SARS epidemic, the COVID-19 outbreak originated during the ‘Spring Festival of China’, which is considered to be one of the most famous traditional festivals in China, during which almost 3 billion people travel countrywide. These circumstances provide favorable conditions for the spread of highly contagious diseases and as such severe difficulties arise in prevention and control of the epidemic. The ‘Spring Festival of China’ took place between January 17 and February 23 in 2003, during which the SARS epidemic was at its peak, while the period of the festival in 2020 was between January 10 and February 18. As it can be noted, there was a rapid rise in COVID-19 cases between 10 and 22 January. Wuhan, the center of the epidemic with 10 million populations, is a central hub in the Spring Festival transportation network. The estimated number of travelers during the 2020 Spring Festival has risen 1.7-fold when compared with the number travelled in 2003, and touched 3.11 billion from 1.82 billion. This large-scale travel traffic might have created favorable conditions for the spread of this dreadful disease [115]. The sequence of events reported for COVID-19 has been summarized in Fig. 2.

**Fig. 3:**
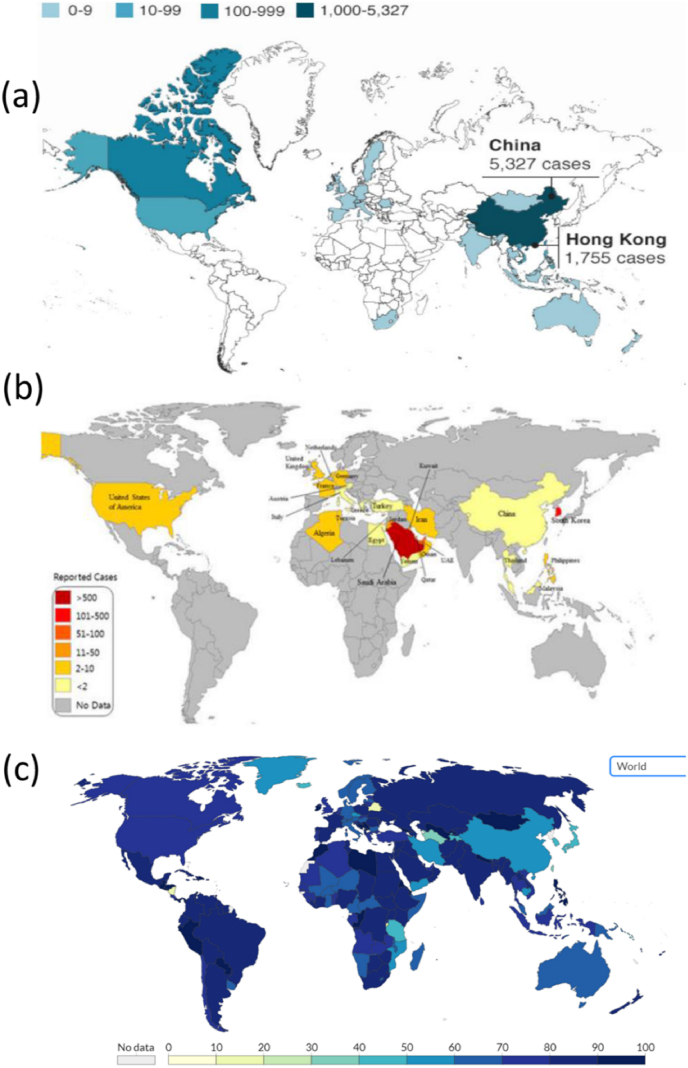
Global distribution maps of various CoVs, a) SARS (Confirmed cases from November 2002 to July 2003); b) MERS (Reported cases from April 2012 to July 2015); c) COVID-19 (Cases reported as on December 7, 2020). Source: SARS-WHO; COVID-19- https://www.bsg.ox.ac.uk/; MERS- [120].

The originating point of the COVID-19 pandemic has been identified to be a 57-year-old female shrimp seller in China's Wuhan city. She was one of the first 27 victims of COVID-19 which has claimed more than 5.15 M lives till now. Wei Guixian, the *‘patient zero’* recovered from the disease in January after one-month-long treatment developed cold symptoms when she was selling shrimps at the Huanan Seafood Market on December 10. After the occurrence, secondary cases started to arise approximately after ten days. Although, the new patients diagnosed had no connection with the marketplace, they had a history of contact with humans there. Reports from infected HCWs in Wuhan confirmed that human-to-human transmission had occurred [116]. The first non-Chinese case of COVID-19 infection was reported from Thailand on January 13, 2020. The case reported being a Chinese tourist who had travelled to Thailand and had no epidemiologic connection with the marketplace [117]. Countries like Italy, Germany, France, Iran, India and US are the worst hit by the COVID-19 pandemic, which has spread to more than 210 countries across the globe. Presently, majority of the countries worldwide are continuing to report an alarming number of COVID-19 cases [118]. In 2020, a global emergency was declared by WHO [119]. According to the latest reports (as on November 8, 2021), total infected cases have climbed to 250,892,192 in which the US has the highest number of infections with 47,345,192 confirmed cases and the highest death toll with 775,346 reported fatalities (WHO). India has the second-highest position with 461, 347 deaths till now. Total number of active cases with deaths and recoveries across the globe has been listed in Table 2. Global distribution of cases reported for SARS, MERS and COVID-19 on its peak can be visualized in Fig. 3.

### 2.4 Virology and pathogenesis of CoVs

Up till the discovery of novel SARS-CoV-2 and its variants, the genomic structure of CoVs was best studied among all RNA viruses. The RNA-based viruses like the CoV or the flu virus have a tendency to mutate around 100 times faster than the DNA-based ones—although the CoV evolves more slowly as compared to influenza viruses [121]. About two-third RNA of CoV encodes viral polymerase (RdRp), RNA synthesis materials, and two large nonstructural polyproteins that are not part of host response modulation (ORF1a-ORF1b); the left over one-third of the genome encodes four structural proteins (spike, S; envelope, E; membrane, M; nucleocapsid, N), and other helper proteins [122]. Though, the length of the CoV genome shows high variability for ORF1a/ORF1b and four structural proteins, it is generally linked with the number and size of accessory proteins (*Coronavirinae in Viral Zone*, available online: https://viralzone. expasy.org/785). The foremost step in virus infection is the interaction of human cells with spike protein of the virus. This is followed by genome encoding which occurs after entering into the cell and this process facilitates the expression of the genes that encode useful accessory proteins. This step is very crucial as it efficiently adapts the CoVs to their human host [115]. Genomic alterations resulting from recombination, gene exchange, gene insertion, or deletion is a common phenomenon among CoVs, and this is expected to occur in future outbreaks as observed in the past epidemics. The CoV subfamily is a rapidly expanding one and with the help of next generation sequencing techniques, the detection and definition of novel CoV species can be improved. In conclusion, CoV classification is continuously altering. Based on the latest classification of the International Committee on Taxonomy of Viruses, there are four genera of thirty-eight unique species [89].

Among the major outbreaks caused by CoVs, palm cats and dromedary camels have been considered to be the natural reservoir for SARS and MERS, respectively [90]. However, innovative virological and genetic studies have shown that bats are reservoir hosts of both SARS-CoV and MERS-CoV and before these viruses spread to humans, they multiply in intermediate hosts. The origin of the newly emerged SARS-CoV-2 is debatable. Few studies revealed that the detected SARS-CoV strains in market civets, were transmitted from horseshoe bats [123,124]. These viruses were found phylogenetically linked to SARS-CoV in bats from China, Europe, Southeast Asia and Africa [84,123,125]. Besides, the genome sequences of SARS-CoV isolated from humans were found to be similar to those in bats. However, some differences were found among the s gene and orf gene, which encode the binding proteins and dispensable protein for replication, respectively [84,125]. Nevertheless, clade2 of S region, ORF and ORF3b in SARS-CoV from bats have maximum variations as compared to SARS-CoV isolated from humans [125,126].

Majority of the strains of MERS-CoV isolated from camels were found to be similar to those obtained from humans, except, genomic variations among S, ORF4b and ORF3 regions [[13], [127]]. The genomic sequencing indicated that MERS-CoVs from humans are phylogenetically allied to those from bats. They have identical genomic and protein structures except for the S proteins [129]. Additionally, the recombination studies of genes encoding ORF1ab and S revealed that MERS-CoV instigated from the exchange of genetic elements between CoVs in camels and bats [128,130]. Due to selective binding with different receptors, MERS-CoV and SARS-CoV differ in their cellular selection for infection [126]. Notably, MERS-CoV utilizes the human dipeptidyl peptidase 4 (DPP4) receptors for cell entry, as compared to angiotensin-converting enzyme 2 receptors used by SARS-CoV [125]. MERS-CoV makes entry into the host through its S protein, a type I transmembrane GP containing 1353 amino acids that exists on the virion surface as a trimer. Consequently, it is recognized by cluster of differentiation 26, CD26 (also known as DPP4); which assists in the infection of the host cells [104] (see Table 3).

The genome sequence of SARS-CoV-2 exhibits close relatedness (88% identity) with two bat-derived SARS-like CoVs (bat- SL-CoVZC45 and bat-SL-CoVZXC21), though its zoonotic source is yet not confirmed. Phylogenetic studies confirmed that SARS-CoV-2 was genetically diverse from SARS-CoV and MERS-CoV. Though, homology modeling analysis shows that both SARS-CoV and SARS-CoV-2 had similar receptor-binding domain structures, regardless of some amino acid variation at significant residues (such as the absence of 8a protein and fluctuation in the number of amino acids in 8b and 3c protein in SARS-CoV-2). In contrast, the main protease is highly conserved between COVID-19 and SARS-CoV with a 96% overall identity [111]. These explanations conclude that bats are the source of origin, while an animal sold at the Wuhan seafood market serves as a missing link (intermediate host), enabling the emergence of the virus in humans. The epidemiology and biological characteristics of SARS, MERS and COVID-19 have been compared in Table 4.

**Table 4:** Epidemiology and clinical characteristics of zoonotic CoVs.

		SARS-CoV	MERS-CoV	COVID-19
**Genus**		β-CoVs, lineage B	β-CoVs, lineage C	β-CoVs, lineage B
Possible natural reservoir		Bat	Bat	Bat
Possible intermediary host		Palm civet	Dromedary camels	Unknown
Origin		Guangdong Province, China	Arabian Peninsula	Hubei Province, China
Clinical Epidemiology	Global cases reported to WHO	>8098	2254 (September 2018)	252, 434, 444 (November 2021)
	Affected countries	29	27	210
	Total number of deaths	916	800	5,092,806
	Fatality rate	>10%	>35%	2–4% (confirmed cases)
	Transmission region	Globally	Regionally	Globally
	Transmission route	----------------Animals-Humans; Humans-Humans----------------		
	Incubation period	2–10 days	2–14 days	1–14 days
	Pandemic potential	Intermediary	Limited	Efficient
	Pandemic containment	Yes	No	No, efforts ongoing
Predominant receptor		Human Angiotensin-Converting enzyme 2 (ACE2)	Human dipeptidyl peptidase 4 (DPP4/CD26)	Human Angiotensin-Converting enzyme 2 (ACE2)
Receptor distribution		Arterial and venous epithelium; arterial smooth muscle; small intestine; respiratory tract epithelium; alveolar monocytes and macrophages	Respiratory tract epithelium; kidney; small intestine; liver and prostate; activated leukocytes	Epithelial cells of the lung; intestine; kidney; heart and blood vessels.
Cell line susceptibility		Respiratory tract; kidney; liver	Respiratory tract; intestinal tract; genitourinary tract; liver; kidney; neurons and monocytes	Respiratory tract; kidney; liver

### 2.5 Transmission and diagnosis of CoV

Transmission of CoV depends on the levels of oral-fecal and respiratory transmission. Based on these, MERS-CoV possesses hard inner and outer shells that facilitate its presence in the surroundings. Hence, MERS-CoV have the highest fecal-oral transmission rate among all CoVs and moderately low respiratory transmission rates [131]. In a patient identified by MERS, the virus was found in his stool samples obtained on days 12 and 16, with a viral load of up to 1031 RNA copies/g [132]. A fecal-oral route of transmission of MERS was not acknowledged in the clinical setting. MERS-CoV seems to be more viable at low temperatures and low humidity. MERS-CoV was recovered after 48 h at 20 °C and 40% relative humidity (RH), and the virus was stable for 8 h at 30 °C and 80% RH and for 24 h at 30 °C and 30% RH [133]. SARS Patients normally show greater virus concentrations and delayed virus excretion in stools. The stool samples of SARS affected individuals are routinely used for virological diagnosis [95].

MERS may be communicated from person-to-person *via* direct contact likely due to droplet transmission. This occurs mostly when there is a close contact such as providing unprotected care to an infected patient. According to the literature, no sustained community transmission for MERS has been reported so far [134]. Studies of family clusters and contacts of patients have reported decreased levels of transmission (i.e., 1%–3%). However, increased transmission has occurred in HCWs because of inadequate infection control measures. Notably, MERS may be transmitted from an asymptomatic source [135].

Airborne transmission of COVID-19 primarily occurs when an infected person coughs or sneezes and the fomites produced, settles in the mouth or nasal mucosa and lungs of the uninfected people who inhaled the infected air; similar to the spread of influenza and other respiratory pathogens. Generally, like most respiratory viruses, it is considered to be most contagious when people are mostly symptomatic. However, cases were also reported from an asymptomatic source in the incubation period of COVID-19. Sufficient data is not available on infectiousness of the disease and research on the newly evolved CoV is still on-going [136].

Similar to SARS and MERS, the diagnosis of COVID-19 infection is based on the history of detailed contacts, travel and precise laboratory testing. Among the various diagnostic platforms available, Real time RT-PCR remains the primary means for diagnosing the new virus strain [137]. This technique is based on RNA isolated from respiratory samples such as oropharyngeal swabs, sputum, nasopharyngeal aspirate, deep tracheal aspirate, or bronchoalveolar lavage. This procedure is advantageous in terms of evaluating the results quickly, revealing the genome structure and viral load [114]. In particular, lower respiratory tract samples shows a significantly higher viral load and genome fraction than upper respiratory tract samples [138]. For MERS, throat swab, sputum, tracheal aspirate or broncho-alveolar lavage specimens are collected and stored at 2–8 °C, and transported within 72 h to the reference laboratories, where they are subjected to a real-time reverse-transcriptase–polymerase-chain-reaction (rRT-PCR) assay [139]. In case of SARS, two different clinical specimens like nasopharyngeal and stool are generally collected to perform RT-PCR [140]. The period and type of specimen collected for RT-PCR play an important role in the diagnosis of COVID-19. It was found that the respiratory samples tested positive for the virus, while for serum, negative results were obtained during the initial incubation phase. Moreover, in the early days of illness, patients have increased viral load despite exhibiting mild symptoms [141].

CRISPR-based assays are also being used to detect novel SARS-CoV-2. The test can be carried out using RNA purified from patient samples, as in qRT-PCR assays. As an alternative technique, the samples can be read out at a faster rate, without requiring elaborate instrumentation. In a very recent work published by Broughton and his co-workers, a rapid (<40 min), easy-to-implement and précised CRISPR–Cas12-based lateral flow assay for detection of SARS-CoV-2 from respiratory swab RNA extracts has been reported. The team validated this method using artificial reference samples and clinical samples from patients in the United States, which included 36 patients infected with COVID-19 and 42 patients compromised with other viral respiratory infections [141].

The clinical spectra of SARS, MERS and COVID-19 infection range from asymptomatic or mild respiratory symptoms to severe acute respiratory illness resulting in respiratory failure, septic shock, multi-organ failure, and sudden death. Children and old age people demonstrate less severe manifestations, with death rarely reported except for significant comorbidities (eg, chronic lung diseases, diabetes) and immune suppression [110]. The clinical manifestation of SARS includes flu-like symptoms with persistent fever and rapidly progressive dyspnea, with 20% of patients suffering from watery diarrhea [92]. The WHO lay down a criterion for ‘‘suspected’’ and ‘‘probable’’ SARS to assist in diagnosis. ‘‘Suspected’’ SARS was defined with symptoms such as high fever and difficulty in breathing/cough. ‘‘Probable’’ SARS was defined as a suspected SARS with respiratory distress syndrome (RDS) or severe pneumonia [99]. Characteristic symptoms of MERS include fever, cough, and shortness of breath. Pneumonia is common but not found in all cases. Gastrointestinal symptoms like vomiting and diarrhea frequently occur [108].

The clinical aspects of COVID-19 were found to be similar to SARS and MERS that caused epidemics in the recent years as in other respiratory infection causing beta-CoVs, The initial symptoms comprising fever, cough, shortness of breath [117]. Although, diarrhea was present in about 20–25% of patients affected with MERS or SARS infection, intestinal symptoms were rarely reported in patients with COVID-19. In another study conducted on 99 patients; additional symptoms like chest pain, confusion, and nausea-vomiting were also found [138].

### 2.6 COVID mutations

**Fig. 4:**
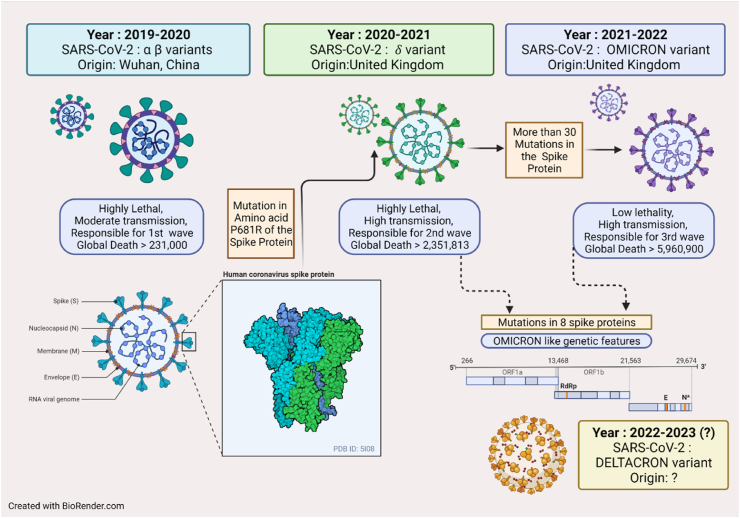
Mutation in COVID from 2019 and their pathogenesis.

COVID has wreaked havoc in the whole world since the last 2 years. Though there has been decline in the number of daily cases, the pandemic is still far from over. The WHO has listed five variants of concerns (VOC) of the coronavirus: Alpha, Beta, Gamma, Delta and Omicron. These variants are more aggressive than the other variants. The Omicron variant is the current dominant strain of the virus and is the major reason behind COVID infection at present. Further, there are two variants of interest: Lambda and Mu (https://timesofindia.indiatimes.com/life-style/health-fitness/health-news/from-delta-to-omicron-symptoms-that-were-common-with-different-variants/photostory/8987611.cms?picid=89687697). The different outbreaks resulting from mutations in the virus protein turned out to be enormously fatal (Fig. 4). The Delta variant, that had 40% more transmission and identified in UK in 2020, had caused a nightmare in India in 2021. A team from Texas University predicted that this mutation alters a single amino acid – P681R - in the SARS-COV-2 spike protein; proline gets replaced by arginine in the furine cleavage site – this results in the heightened infectivity of the virus. They found that in cultured human-airway epithelial cells infected with equal numbers of Delta and Alpha viral particles, Delta rapidly outcompeted the Alpha variant and this trend was observed globally [[142], [143]]. The Omicron variant (B.1.1.529), found in South Africa, was also reported as a variant of concern by WHO. Researchers reported that the variant undergoes more than 30 mutations in the spike protein that allows targeting the host cells. Because of such a large number of mutations in the spike protein, the virus tends to evade the antibodies generated by vaccination. News of another super variant has emerged recently; however, WHO has said that there is nothing called “Deltacron”! The ‘Deltacron’ sequences were generated from virus samples obtained by a group of scientists in the University of Cyprus in December to track the spread of SARS-CoV-2 variants in Cyprus; they found an Omicron-like genetic signature in the gene for the spike protein, which helps the virus to enter cells. They further hypothesized that some Delta virus particles had independently evolved mutations in the spike gene similar to those common in Omicron. To this, a virologist anonymously commented that in the age of social media and rapid exchange of information, scientists need to be very careful with their words [145]. However, the exchange to information in the scientific community, regarding the virus mutations in the midst of pandemic facilitates for better preparation by the medical and scientific world is necessary.

Amidst all this, the world is witnessing another round of surge in COVID cases in UK, Japan, and China – the Chinese city of Shanghai being the worst affected (https://www.cnbc.com/2022/04/12/new-omicron-xe-variant-detected-in-japan-as-uk-cases-rise-.html). It is assumed that an Omicron sub-variant XE, a mix of two Omicron strains BA.1 and BA.2 is the causative agent this time. While China is struggling to contain the spread of virus among its masses (https://swarajyamag.com/world/covid-in-china-shanghai-witnesses-record-surge-in-covid-cases-amid-growing-public-anger), this strain has still not reached most parts of the world at present [143,144]; the future is still at risk and no one should let their guard down!

### 2.7 Management and treatment of CoVs

Different strategies have been proposed to treat infections caused by MERS-CoV and SARS-CoV, comprising the use of interferons (IFNs), antibodies, inhibitors of viral and host proteases and host-directed therapies [146]. Patients generally receive supportive care, which is usually accompanied by several combinations of drugs in the absence of a clinically proven, effective antiviral therapy. Ribavirin and various kinds of IFN have been administered to patients with MERS in Saudi Arabia and China, mostly in combination with a broad-spectrum antibiotic and oxygen [147]. However, the effectiveness of these treatments for SARS and MERS infection is still imprecise. Moreover, treatment for MERS normally starts in a later stage, when immunopathology comes into role and antiviral drugs are ineffective. During the SARS outbreak, Ribavirin was often used in combination with corticosteroids that are known to have an anti-inflammatory effect [146]. Even though IFNs are effective against MERS-CoV *in vitro* [148], their effect in humans is yet to be proved. Interestingly, the protease inhibitors, lopinavir and ritonavir, which are used in combination to treat HIV infection, improved the condition of SARS patients when combined with ribavirin, compared to patients who were treated with ribavirin alone [149]. Though, lopinavir did not show any antiviral activity against MERS-CoV *in vitro* [145]. Two independent research groups reported that both chloroquine and chlorpromazine have potential therapeutic antiviral activity. Chlorpromazine mainly affects the assembly of clathrin-coated pits at the plasma membrane, while chloroquine plays an important role in endosomal acidification. Eventually, these drugs constrain MERS-CoV endocytosis at the host cell membrane and prevents fusion of endosomes intracellularly by interrupting the above mentioned biological processes [150,151].

Vaccines are one of the preventive measures against infection and disease occurrence when exposed to the specific pathogen of interest, mainly in populations with severe comorbidities. Effective vaccines are essential to prevent and control sporadic viral attacks and emerging virus-mediated epidemics, such as the recent COVID-19 outbreak caused by SARS-CoV-2. Nevertheless, there are always some social, economic and clinical hurdles in vaccine administration and vaccination programmes such as, (a) the readiness of the public to undertake a novel vaccine, (b) the side effects and adverse reactions of vaccination, (c) the potential difference and/or low efficacy of the vaccine in populations of different clinical trials, and lastly (d) the accessibility of the vaccines to a specific population (including the cost and availability of the vaccine).

In the context of the current COVID-19 outbreak, vaccines will help control and reduce disease transmission by creating herd immunity along with protecting healthy individuals from infection, along with evidence based management [82,152]. There are already several vaccines available and some of potential ones are in line of development. The vaccines available to date and in development phase include viral vector-based vaccine, DNA vaccine, inactivated whole-virus vaccine, live attenuated vaccine, subunit vaccine and virus-like particles (VLP) based vaccine. Although, SARS was fully contained during 2003, and MERS has been controlled from causing high mortalities, the newly emerged COVID-19 is spreading rapidly with a significant increase in the number of cases and fatalities every passing day. Broad-spectrum vaccines effective against different variants are urgently required to prevent COVID-19 from spreading and causing other pandemics in future.

Live-attenuated vaccines, designed for SARS, may be evaluated for COVID-19 affected patients. Moreover, rhesus theta-defensin and protein cage nanoparticles are known to be innate immunomodulators with high anti-SARS efficiency [153,154]. Due to the higher similarities and close phylogenetic relationship between SARS-CoV and SARS-CoV-2, protein cage nanoparticles designed for SARS disease can be evaluated for COVID-19. Due to the urgency of the current scenario of COVID-19 outbreak, vaccination strategies based on viral vectors, recombinant protein, and VLPs, which have been developed or are being developed for SARS and/or MERS can be revised for utilization against COVID-19 [155].

Vaccines can be applied in particular risk groups. Different approaches have been employed for the development of MERS vaccines [82]. An orthopoxviral-based vaccine, employing Modified Vaccinia Ankara (MVA) that expresses the spike protein, has been developed and shown to induce neutralizing antibodies and specific cytotoxic T cell response against MERS-CoV [156]. Moreover, the MVA has been validated as an effective vaccine platform in humans [157]. Unlike prophylactic regimens, protection induced by vaccination is long lasting, although the effect is not immediate. Thus, vaccination would be more appropriate for camel shepherds and slaughterhouse workers who are at a greater risk of being infected by MERS-CoV.

As of December 2020, over 100 countries were working together to find effective therapeutics as soon as possible, via 614 trials which have been registered under solidarity trial works (WHO; https://www.who.int/emergencies/diseases/novel-coronavirus-2019/global-research-on-novel-coronavirus-2019-ncov/solidarity-clinical-trial-for-covid-19-treatments). It was assumed to take 3 more months for most candidates to start phase 3 clinical trials except for those funded by Coalition for Epidemic Preparedness Innovations (CEPI). Around 9 clinical trials were registered under the Clinical Trials Registry (ClinicalTrials.gov) for COVID-19 therapeutics, of which 5 studies hydroxychloroquine, combination of lopinavir with ritonavir and arbidol, mesenchymal stem cells, traditional Chinese medicine and glucocorticoid therapy usage. The remaining 4 studies include investigation of antivirals; interferons; darunavir and cobicistat; arbidol and remdesivir usage for patients [141], which subsequently were licensed.

The New Year, 2021, brought fresh hopes with the licensing of the first vaccine against COVID 19 by Pfizer-BioNTech; it is an mRNA vaccine without live virus, but containing a small portion of viral sequence of the SARS-COV-2 virus that would instruct the vaccinated person to produce the spike protein displayed on the virus coat. This protein then triggers the immune response and subsequently prevents viral replication. However, potential side effects of this vaccine include nausea, malaise, muscle and joint pains, chills, fever, injection site pain and swelling, fatigue etc. (https://www.pfizer.com/news/hot-topics/the_facts_about_pfizer_and_biontech_s_covid_19_vaccine). India also licensed 2 vaccines, Covishield (the local name for the Oxford-AstraZeneca vaccine developed in the UK and manufactured by the Serum Institute of India) (https://www.seruminstitute.com/product_covishield.php) and Covaxin, locally-made by pharma company Bharat Biotech (https://www.bharatbiotech.com/covaxin.html). The Oxford-AstraZeneca vaccine is being manufactured locally by the Serum Institute of India, the world's largest vaccine manufacturer. This vaccine is prepared from a weakened version of a common cold virus (known as an adenovirus) isolated from chimpanzees. It has been modified to resemble coronavirus - although it cannot cause illness. Some of the first doses of Covishield have been already shipped from India to Bhutan, Maldives, Bangladesh, Nepal, Myanmar and Seychelles - some in the form of “gifts” and the rest in line with commercial agreements. A large number of vaccines are still underway, including Sputnik V from Russia.

Despite the current progress, extensive work is needed to develop and streamline safe and effective vaccines for individuals at high risk of COVID-19 pandemic in order to control the ongoing situation and future risk of evolving variants. Despite all this, the socio-economic impact of the pandemic has been on the downside [158] – the long duration would be required for this recovery!

## 3 Critical discussion ON HOW to contain a pandemic

Lessons learned from the MERS and SARS outbreaks can deliver valuable insight into how the current epidemic and emerging viral respiratory infections can be controlled. Some of the prompt actions include: early case detection, isolation of suspected cases, effective contact tracing, practicing hand hygiene and preventing direct contact with suspected animal reservoir host etc. [159]. Unfortunately, some lessons were not observed as transparency was missing between the government officials and the public, which in turn led to the rapid spread of COVID-19 in 2020.

In a study by Wu and his co-workers, a mathematical model using a combination of household-based quarantine, isolation of cases outside the household, and targeted prophylactic use proved to be effective in the prevention of viral spread [160]. An extent of more than 3 days in contact tracing, case identification, and quarantine adversely affected the efficacy of isolation later on [108]. The delay in the isolation of patients in the healthcare setting also led to the rapid transmission of SARS [161]. Furthermore, the Al-Hasa MERS-CoV outbreak and rise of MERS-CoV cases in April–May 2014 indicate prevalence of inadequate infection control measures [162].

Most importantly, prompt identification of patients with SARS along with the implementation of infection control measures resulted in prevention of secondary transmission of SARS [163]. Different strategies may be acquired for the control of an outbreak in a healthcare setting. If the outbreak is detected late, abrupt hospital closure is required to control nosocomial transmission, or early closure in an outbreak is appropriate to take out exposed persons to a designated location or distancing them in a separate place [164]. Thus, prompt identification of cases with proper isolation of infected individuals in properly ventilated hospitals (negative pressure rooms) in the HCWs or community quarantine is a strategic measure to prevent the spread of emerging respiratory pathogens.

R_o_ is defined as the basic reproductive number of an infectious disease. It represents the number of new infections estimate to stem from a single case. The larger the R_o_, the more difficult is to control a pandemic; hence control measures aim to reduce the reproductive number to less than 1. In general, an epidemic will increase as long as R_o_ is greater than 1. It has been estimated that in case of the novel SARS-CoV-2, the R_o_ is 2.2 approximately, which implies that on an average, each patient has been spreading infection to 2.2 other people [116]. The R_o_ of SARS-CoV was estimated to be around 3 and consecutive SARS outbreaks were effectively controlled by isolation of patients and careful infection control [165].

## 4 Conclusions

In December 2019, the seventh CoV according to classification i.e., SARS-CoV-2 emerged in Wuhan, Hubei Province of China. Progressively, it spread to 210 countries worldwide causing COVID-19 and became another severe pandemic caused by an RNA virus, after SARS and MERS. Since there is no definite treatment for CoVs, there is an urgent need for global surveillance of people infected with COVID-19. However, further efforts to understand COVID-19 pathogenesis in a much better way are needed; this would help to identify the viral and host factors that play a major role in the transmission of COVID-19 in humans and thus and offer possible treatment. Presently, we have to enforce strict preventive and control measures that reduce the risk of disease transmission until the developed vaccines are broadly effective or an antiviral agent is successful. Accomplishments in the development of vaccines and therapeutic agents for SARS-CoV and MERS-CoV can give a ray of hope to ease the development of effective vaccines and therapeutics against COVID-19. Future research should be focused towards the study of SARS-CoV-2 in appropriate animal models for investigating the replication, transmission, disease manifestations and pathogenesis associated with experimental inoculation of the emerging virus. Last but not the least; effective measures should be employed to avoid the unpredictable risk and possibility of a local outbreak turning into a global pandemic.

## Ethical approval

This article does not require any human/animal subjects to acquire such approval.

## Source of funding

This study received no specific grant from any funding agency in the public, commercial, or not-for-profit sectors.

## Author contributions

**Ishani Chakrabartty:** Conceptualization, Data curation, Writing-Original draft preparation, Writing- Reviewing and Editing. **Maryam Khan:** Conceptualization, Data curation, Writing-Original draft preparation, Writing- Reviewing and Editing. **Saurov Mahanta:** Data curation, Writing-Original draft preparation, Writing- Reviewing and Editing. **Hitesh Chopra:** Data curation, Writing-Original draft preparation, Writing- Reviewing and Editing. **Manish Dhawan:** Data curation, Writing-Original draft preparation, Writing- Reviewing and Editing. **Om Prakash Choudhary:** Data curation, Writing- Reviewing and Editing. **Shabana Bibi:** Writing- Reviewing and Editing. **Yugal Kishore Mohanta:** Conceptualization, Writing-Reviewing and Editing, Visualization. **Talha Bin Emran:** Conceptualization, Writing-Reviewing and Editing, Visualization.

## Trial registry number

1. Name of the registry: Not applicable.
2. Unique Identifying number or registration ID:Not applicable.
3. Hyperlink to your specific registration (must be publicly accessible and will be checked): Not applicable.

## Guarantor

Talha Bin Emran, Ph.D., Associate Professor, Department of Pharmacy, BGC Trust University Bangladesh, Chittagong 4381, Bangladesh. T: +88-030-3356193, Fax: +88-031-2550224, Cell: +88–01819942214. https://orcid.org/0000-0003-3188-2272. E-mail: talhabmb@bgctub.ac.bd.

## Data availability statement

The data that support the findings of this study are available from the corresponding author upon reasonable request.

## Ethics approval and consent to participate

Not applicable.

## Consent for publication

Not applicable.

## Declaration of competing interest

The authors declare that they have no conflict of interest to disclose.

## LIST OF ABBREVIATIONS

COVID-19: Coronavirus disease 2019
DRC: Democratic Republic of the Congo
DPP4: dipeptidyl peptidase 4
EBOV: Ebola virus
EVD: Ebola virus disease
EBOLA: Ebola Virus disease
INFs: Interferons
MERS: Middle East respiratory syndrome
RH: Relative humidity
rRT-PCR: Reverse-transcriptase–polymerase-chain-reaction
SARS: Severe acute respiratory syndrome
SARS-CoV-2: Severe acute respiratory syndrome coronavirus 2
ZIKA: GP: Viral envelope glycoprotein
VP: viral matrix proteins
VLP: Virus like particles based vaccine
WHO: World Health Organization
ZKV: Zika virus disease
